# (*E*)-1-(4-Amino­phen­yl)-3-(2-chloro­phen­yl)prop-2-en-1-one

**DOI:** 10.1107/S1600536808030456

**Published:** 2008-09-27

**Authors:** Hoong-Kun Fun, Reza Kia, P. S. Patil, S. M. Dharmaprakash, Ibrahim Abdul Razak

**Affiliations:** a X-ray Crystallography Unit, School of Physics, Universiti Sains Malaysia, 11800 USM, Penang, Malaysia; b Department of Physics, K. L. E. Society’s K. L. E. Institute of Technology, Gokul Road, Hubli 590 030, India

## Abstract

The title compound, C_15_H_12_ClNO, a substituted chalcone, adopts an *E* configuration with respect to the C=C bond of the enone unit. The mol­ecule is not planar, as can be seen from the dihedral angle of 28.9 (2)° between the two rings which are twisted from each other. The enone segment of the mol­ecule is not coplanar with the chloro­phenyl ring, making a dihedral angle of 23.4 (3)° with it. The amino group is also not coplanar with the ring to which it is bound, making a dihedral angle of 35 (4)°. In the crystal structure, adjacent mol­ecules are linked by N—H⋯O inter­actions into one-dimensional infinite chains along the *c* axis, and are further stacked as one-dimensional zigzag chains down the *b* axis, forming two-dimensional extended networks parallel to the *bc* plane.

## Related literature

For related literature on hydrogen-bond motifs, see Bernstein *et al.* (1995 ▶). For bond-length data, see Allen *et al.* (1987 ▶). For related structures, see, for example: Patil *et al.* (2007*a* ▶,*b* ▶,*c* ▶). For background to the applications of substituted chalcones, see, for example: Agrinskaya *et al.* (1999 ▶); Gu *et al.* (2008*a* ▶,*b* ▶,*c* ▶).
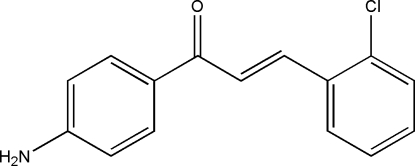


## Experimental

### 

#### Crystal data

C_15_H_12_ClNO
*M* *_r_* = 257.71
Monoclinic, 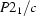

*a* = 22.4670 (19) Å
*b* = 3.9254 (3) Å
*c* = 14.5796 (11) Å
β = 107.944 (6)°
*V* = 1223.26 (17) Å^3^
*Z* = 4
Mo *K*α radiation
μ = 0.30 mm^−1^
*T* = 100.0 (1) K
0.28 × 0.27 × 0.06 mm

#### Data collection

Bruker SMART APEXII CCD area-detector diffractometer
Absorption correction: multi-scan (**SADABS**; Bruker, 2005 ▶) *T*_min_ = 0.935, *T*_max_ = 0.985
12218 measured reflections
2509 independent reflections
1717 reflections with *I* > 2σ(*I*)
*R*_int_ = 0.089

#### Refinement

*R*[*F* ^2^ > 2σ(*F* ^2^)] = 0.072
*wR*(*F* ^2^) = 0.190
*S* = 1.13
2509 reflections
171 parameters
H atoms treated by a mixture of independent and constrained refinement
Δρ_max_ = 0.41 e Å^−3^
Δρ_min_ = −0.40 e Å^−3^

### 

Data collection: *APEX2* (Bruker, 2005 ▶); cell refinement: *APEX2*; data reduction: *SAINT* (Bruker, 2005 ▶); program(s) used to solve structure: *SHELXTL* (Sheldrick, 2008 ▶); program(s) used to refine structure: *SHELXTL*; molecular graphics: *SHELXTL*; software used to prepare material for publication: *SHELXTL*, *PARST* (Nardelli, 1995 ▶) and *PLATON* (Spek, 2003 ▶).

## Supplementary Material

Crystal structure: contains datablocks global, I. DOI: 10.1107/S1600536808030456/cs2093sup1.cif

Structure factors: contains datablocks I. DOI: 10.1107/S1600536808030456/cs2093Isup2.hkl

Additional supplementary materials:  crystallographic information; 3D view; checkCIF report

## Figures and Tables

**Table 1:** Hydrogen-bond geometry (Å, °)

*D*—H⋯*A*	*D*—H	H⋯*A*	*D*⋯*A*	*D*—H⋯*A*
N1—H1N1⋯N1^i^	0.85 (4)	2.30 (5)	3.098 (6)	158 (4)
N1—H2N1⋯O1^ii^	0.83 (4)	2.10 (4)	2.923 (5)	171 (4)

Symmetry codes: (i) 
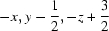
; (ii) 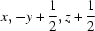.

**Fig. 1.**
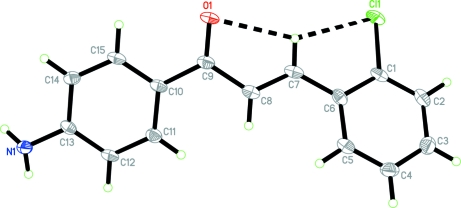
The molecular structure of the title compound, showing 50% probability displacement ellipsoids and the atomic numbering. Intramolecular interactions are drawn as dashed lines.

**Fig. 2.**
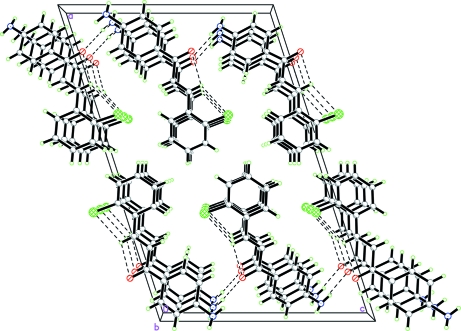
The crystal packing of (I), viewed down the b-axis, showing 1-D extended chains along the c-axis, and stacking of these chains along the b-axis. Intramolecular and intermolecular interactions are drawn as dashed lines.

**Fig. 3.**
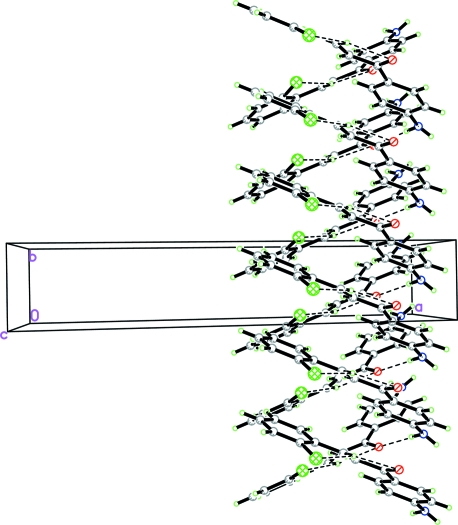
The crystal packing of (I), viewed down the c-axis, showing 1-D zigzag chains along the b-axis.

## supplementary crystallographic information

### Comment

Chalcone derivatives exhibit fascinating nonlinear optical properties (Agrinskaya *et al.*, 1999). Among the nonlinear optical applications, optical limiting (OL) has been particularly promising (Gu *et al.*, 2008a; Gu, *et al.*, 2008c). Optical limiters are devices that strongly attenuate intense optical beams while exhibiting high transmittance for low-intensity ambient light levels. This behavior has applications such as the protection of human eyes and optically sensitive equipments. Chalcone derivatives are very good candidates for optical limiting applications (Gu *et al.*, 2008b). As a part of our crystallographic studies (Patil *et al.*, 2007a; Patil *et al.*, 2007b; Patil *et al.*, 2007c), the title compound was synthesized and its crystal structure is reported.

The title compound (I, Fig 1), a substituted chalcone, adopts an *E* configuration with respect to the C═C bond of the enone unit. Intramolecular C—H···O and C—H···Cl hydrogen bonds involving the enone group generate *S*(5) ring motifs (Bernstein *et al.*, 1995). The bond lengths are within the normal ranges (Allen *et al.*, 1987). The molecule is not planar, with a maximum deviation from the mean plane of C1–C15/N1/O1 being -1.135 (3) Å for atom Cl1. The amino group is also not coplanar with the phenyl ring to which it is bound. The dihedral angle between the C10–C15 ring and the N1-H1N1-H2N1 plane of the NH_2_ group is 34.7(3.7)°. The two phenyl rings are twisted to each other with the dihedral angle of 28.9 (2)°. Adjacent molecules are linked together by N—H···O interactions (Table 1) into 1-D infinite chains along the *c* axis in the crystal structure (Fig. 2). Such chains are further stacked as 1-D zigzag chains down the *b*-axis (Fig. 3), too, to form 2-D extended networks parallel to the *bc* plane.

### Experimental

The compound (I) was synthesized by the condensation of 2-chlorobenzaldehyde (0.01 mol, 1.13 g) with *p*-aminoacetophenone (0.01 mol, 1.35 g) in methanol (60 ml) in the presence of a catalytic amount of sodium hydroxide solution (5 ml, 30°). After stirring (6 h), the contents of the flask were poured into ice-cold water (500 ml) and left to stand for 5 h. The resulting crude solid was filtered and dried. Crystals suitable for *X*-ray analysis were grown by slow evaporation of an acetone solution at room temperature.

### Refinement

The hydrogen atoms of the amino group were located from the difference Fourier map and refined freely. The remaining H atoms were located in the riding model approximation with C—H= 0.93 Å, and with *U*_iso_(H) = 1.2*U*_eq_(C).

### Crystal data

**Table:**

C_15_H_12_ClNO	*F*(000) = 536
*M**_r_* = 257.71	*D*_x_ = 1.399 Mg m^−^^3^
Monoclinic, *P*2_1_/*c*	Mo *K*α radiation, λ = 0.71073 Å
Hall symbol: -P 2ybc	Cell parameters from 2522 reflections
*a* = 22.4670 (19) Å	θ = 2.9–30.2°
*b* = 3.9254 (3) Å	µ = 0.30 mm^−^^1^
*c* = 14.5796 (11) Å	*T* = 100 K
β = 107.944 (6)°	Plate, yellow
*V* = 1223.26 (17) Å^3^	0.28 × 0.27 × 0.06 mm
*Z* = 4	

### Data collection

**Table:**

Bruker SMART APEXII CCD area-detector diffractometer	2509 independent reflections
Radiation source: fine-focus sealed tube	1717 reflections with *I* > 2σ(*I*)
graphite	*R*_int_ = 0.090
φ and ω scans	θ_max_ = 26.5°, θ_min_ = 1.0°
Absorption correction: multi-scan (*SADABS*; Bruker, 2005)	*h* = −28→28
*T*_min_ = 0.935, *T*_max_ = 0.985	*k* = −4→4
12218 measured reflections	*l* = −18→18

### Refinement

**Table:**

Refinement on *F*^2^	Primary atom site location: structure-invariant direct methods
Least-squares matrix: full	Secondary atom site location: difference Fourier map
*R*[*F*^2^ > 2σ(*F*^2^)] = 0.072	Hydrogen site location: inferred from neighbouring sites
*wR*(*F*^2^) = 0.190	H atoms treated by a mixture of independent and constrained refinement
*S* = 1.13	*w* = 1/[σ^2^(*F*_o_^2^) + (0.0752*P*)^2^ + 2.4541*P*] where *P* = (*F*_o_^2^ + 2*F*_c_^2^)/3
2509 reflections	(Δ/σ)_max_ < 0.001
171 parameters	Δρ_max_ = 0.41 e Å^−^^3^
0 restraints	Δρ_min_ = −0.40 e Å^−^^3^

### Special details

**Table:**

Experimental. The low-temperature data was collected with the Oxford Cyrosystem Cobra low-temperature attachment.

### Fractional atomic coordinates and isotropic or equivalent isotropic displacement parameters (Å^2^)

**Table:**

	*x*	*y*	*z*	*U*_iso_*/*U*_eq_	
Cl1	0.34916 (5)	0.9235 (3)	0.37527 (7)	0.0286 (3)	
O1	0.15206 (14)	0.7004 (9)	0.45043 (19)	0.0269 (8)	
N1	0.05603 (19)	0.0034 (9)	0.7731 (3)	0.0191 (8)	
C1	0.3762 (2)	1.0766 (12)	0.4936 (3)	0.0209 (9)	
C2	0.4360 (2)	1.2147 (12)	0.5233 (3)	0.0253 (10)	
H2A	0.4594	1.2283	0.4807	0.030*	
C3	0.4604 (2)	1.3321 (12)	0.6171 (3)	0.0252 (10)	
H3A	0.5006	1.4230	0.6383	0.030*	
C4	0.4246 (2)	1.3131 (12)	0.6790 (3)	0.0249 (10)	
H4A	0.4407	1.3939	0.7417	0.030*	
C5	0.3654 (2)	1.1761 (12)	0.6487 (3)	0.0224 (10)	
H5A	0.3422	1.1667	0.6915	0.027*	
C6	0.33907 (19)	1.0500 (11)	0.5547 (3)	0.0190 (9)	
C7	0.2757 (2)	0.9108 (11)	0.5222 (3)	0.0209 (9)	
H7A	0.2566	0.8946	0.4560	0.025*	
C8	0.24290 (19)	0.8055 (11)	0.5784 (3)	0.0206 (10)	
H8A	0.2606	0.8178	0.6450	0.025*	
C9	0.1783 (2)	0.6678 (11)	0.5372 (3)	0.0199 (9)	
C10	0.14809 (19)	0.4951 (11)	0.6006 (3)	0.0180 (9)	
C11	0.1771 (2)	0.4548 (11)	0.7004 (3)	0.0195 (9)	
H11A	0.2172	0.5414	0.7284	0.023*	
C12	0.14740 (19)	0.2898 (11)	0.7572 (3)	0.0199 (9)	
H12A	0.1675	0.2655	0.8229	0.024*	
C13	0.08702 (19)	0.1580 (10)	0.7165 (3)	0.0169 (9)	
C14	0.0572 (2)	0.2036 (11)	0.6173 (3)	0.0187 (9)	
H14A	0.0168	0.1229	0.5892	0.022*	
C15	0.08799 (19)	0.3678 (11)	0.5619 (3)	0.0198 (10)	
H15A	0.0678	0.3944	0.4962	0.024*	
H1N1	0.027 (2)	−0.133 (12)	0.745 (3)	0.010 (11)*	
H2N1	0.081 (2)	−0.044 (11)	0.827 (3)	0.016 (11)*	

### Atomic displacement parameters (Å^2^)

**Table:**

	*U*^11^	*U*^22^	*U*^33^	*U*^12^	*U*^13^	*U*^23^
Cl1	0.0359 (7)	0.0389 (7)	0.0140 (5)	−0.0011 (6)	0.0120 (4)	0.0004 (5)
O1	0.0299 (17)	0.040 (2)	0.0094 (14)	−0.0045 (15)	0.0036 (12)	0.0015 (13)
N1	0.020 (2)	0.022 (2)	0.0140 (18)	−0.0022 (17)	0.0038 (16)	0.0004 (16)
C1	0.030 (2)	0.021 (2)	0.0133 (18)	0.004 (2)	0.0085 (17)	0.0038 (18)
C2	0.029 (2)	0.027 (3)	0.023 (2)	0.001 (2)	0.0129 (19)	0.0079 (19)
C3	0.022 (2)	0.024 (3)	0.027 (2)	−0.0013 (19)	0.0033 (19)	0.0067 (19)
C4	0.032 (3)	0.024 (3)	0.016 (2)	−0.002 (2)	0.0024 (18)	0.0014 (18)
C5	0.027 (2)	0.025 (3)	0.016 (2)	0.003 (2)	0.0082 (17)	0.0047 (18)
C6	0.026 (2)	0.014 (2)	0.0195 (19)	0.0019 (18)	0.0104 (17)	0.0034 (17)
C7	0.026 (2)	0.024 (2)	0.0122 (18)	0.006 (2)	0.0053 (16)	0.0012 (18)
C8	0.026 (2)	0.023 (2)	0.0130 (19)	0.0019 (19)	0.0060 (17)	0.0011 (17)
C9	0.023 (2)	0.024 (2)	0.0130 (19)	0.0029 (19)	0.0054 (17)	−0.0012 (17)
C10	0.023 (2)	0.020 (2)	0.0116 (18)	0.0030 (18)	0.0066 (16)	−0.0019 (16)
C11	0.021 (2)	0.023 (2)	0.0160 (19)	0.0023 (19)	0.0072 (16)	−0.0017 (18)
C12	0.026 (2)	0.023 (2)	0.0107 (18)	0.0020 (19)	0.0061 (17)	−0.0018 (17)
C13	0.023 (2)	0.015 (2)	0.0150 (19)	0.0039 (18)	0.0097 (16)	−0.0022 (16)
C14	0.021 (2)	0.019 (2)	0.0153 (19)	0.0000 (18)	0.0036 (16)	−0.0037 (17)
C15	0.025 (2)	0.023 (3)	0.0108 (18)	0.0059 (19)	0.0052 (16)	0.0012 (17)

### Geometric parameters (Å, °)

**Table:**

Cl1—C1	1.749 (4)	C7—C8	1.325 (6)
O1—C9	1.226 (5)	C7—H7A	0.9300
N1—C13	1.374 (5)	C8—C9	1.491 (6)
N1—H1N1	0.84 (5)	C8—H8A	0.9300
N1—H2N1	0.83 (5)	C9—C10	1.471 (6)
C1—C2	1.389 (6)	C10—C15	1.386 (6)
C1—C6	1.400 (5)	C10—C11	1.408 (5)
C2—C3	1.387 (6)	C11—C12	1.376 (6)
C2—H2A	0.9300	C11—H11A	0.9300
C3—C4	1.384 (6)	C12—C13	1.401 (6)
C3—H3A	0.9300	C12—H12A	0.9300
C4—C5	1.375 (6)	C13—C14	1.405 (5)
C4—H4A	0.9300	C14—C15	1.375 (6)
C5—C6	1.404 (6)	C14—H14A	0.9300
C5—H5A	0.9300	C15—H15A	0.9300
C6—C7	1.461 (6)		
			
C13—N1—H1N1	116 (3)	C7—C8—C9	121.4 (4)
C13—N1—H2N1	111 (3)	C7—C8—H8A	119.3
H1N1—N1—H2N1	120 (4)	C9—C8—H8A	119.3
C2—C1—C6	122.7 (4)	O1—C9—C10	121.9 (4)
C2—C1—Cl1	116.8 (3)	O1—C9—C8	118.5 (4)
C6—C1—Cl1	120.6 (3)	C10—C9—C8	119.6 (3)
C3—C2—C1	119.2 (4)	C15—C10—C11	117.6 (4)
C3—C2—H2A	120.4	C15—C10—C9	119.3 (3)
C1—C2—H2A	120.4	C11—C10—C9	123.0 (4)
C4—C3—C2	119.5 (4)	C12—C11—C10	121.2 (4)
C4—C3—H3A	120.3	C12—C11—H11A	119.4
C2—C3—H3A	120.3	C10—C11—H11A	119.4
C5—C4—C3	120.7 (4)	C11—C12—C13	120.4 (4)
C5—C4—H4A	119.6	C11—C12—H12A	119.8
C3—C4—H4A	119.6	C13—C12—H12A	119.8
C4—C5—C6	121.7 (4)	N1—C13—C12	120.8 (4)
C4—C5—H5A	119.1	N1—C13—C14	120.4 (4)
C6—C5—H5A	119.1	C12—C13—C14	118.7 (4)
C1—C6—C5	116.1 (4)	C15—C14—C13	119.9 (4)
C1—C6—C7	122.2 (4)	C15—C14—H14A	120.0
C5—C6—C7	121.6 (4)	C13—C14—H14A	120.0
C8—C7—C6	126.0 (4)	C14—C15—C10	122.2 (4)
C8—C7—H7A	117.0	C14—C15—H15A	118.9
C6—C7—H7A	117.0	C10—C15—H15A	118.9
			
C6—C1—C2—C3	0.0 (7)	C7—C8—C9—C10	168.6 (4)
Cl1—C1—C2—C3	−178.4 (4)	O1—C9—C10—C15	−0.6 (6)
C1—C2—C3—C4	−0.7 (7)	C8—C9—C10—C15	179.9 (4)
C2—C3—C4—C5	0.7 (7)	O1—C9—C10—C11	−179.9 (4)
C3—C4—C5—C6	0.2 (7)	C8—C9—C10—C11	0.7 (6)
C2—C1—C6—C5	0.8 (6)	C15—C10—C11—C12	1.1 (6)
Cl1—C1—C6—C5	179.1 (3)	C9—C10—C11—C12	−179.6 (4)
C2—C1—C6—C7	178.7 (4)	C10—C11—C12—C13	−0.1 (6)
Cl1—C1—C6—C7	−3.0 (6)	C11—C12—C13—N1	−177.7 (4)
C4—C5—C6—C1	−0.9 (6)	C11—C12—C13—C14	−1.2 (6)
C4—C5—C6—C7	−178.8 (4)	N1—C13—C14—C15	178.0 (4)
C1—C6—C7—C8	163.8 (5)	C12—C13—C14—C15	1.5 (6)
C5—C6—C7—C8	−18.5 (7)	C13—C14—C15—C10	−0.5 (7)
C6—C7—C8—C9	179.9 (4)	C11—C10—C15—C14	−0.8 (6)
C7—C8—C9—O1	−10.9 (7)	C9—C10—C15—C14	179.9 (4)

### Hydrogen-bond geometry (Å, °)

**Table:**

*D*—H···*A*	*D*—H	H···*A*	*D*···*A*	*D*—H···*A*
N1—H1N1···N1^i^	0.85 (4)	2.30 (5)	3.098 (6)	158 (4)
N1—H2N1···O1^ii^	0.83 (4)	2.10 (4)	2.923 (5)	171 (4)
C7—H7A···Cl1	0.93	2.69	3.081 (5)	106.
C7—H7A···O1	0.93	2.45	2.775 (6)	101.

Symmetry codes: (i) −*x*, *y*−1/2, −*z*+3/2; (ii) *x*, −*y*+1/2, *z*+1/2.
